# Hippocampal volume asymmetry in Alzheimer disease: A systematic review and meta-analysis

**DOI:** 10.1097/MD.0000000000041662

**Published:** 2025-03-07

**Authors:** Yori Pusparani, Chih-Yang Lin, Yih-Kuen Jan, Fu-Yu Lin, Ben-Yi Liau, John Sahaya Rani Alex, Jeetashree Aparajeeta, Wen-Hung Chao, Chi-Wen Lung

**Affiliations:** a Department of Visual Communication Design, Budi Luhur University, Jakarta, Indonesia; b Department of Digital Media Design, Asia University, Taichung, Taiwan; c Department of Mechanical Engineering, National Central University, Taoyuan, Taiwan; d Rehabilitation Engineering Lab, Department of Health and Kinesiology, University of Illinois at Urbana-Champaign, Urbana, IL; e Department of Neurology, China Medical University Hospital, Taichung, Taiwan; f Department of Automatic Control Engineering, Feng Chia University, Taichung, Taiwan; g School of Electronics Engineering, Vellore Institute of Technology, Chennai, India; h Department of Creative Product Design, Asia University, Taichung, Taiwan.

**Keywords:** asymmetry, automatic, manual, segmentation, volume reduction

## Abstract

**Background:**

Biomarkers play an important role in the diagnosis of early-stage Alzheimer disease (AD), with the hippocampal emerging as the most reliable indicator of AD pathology. Elucidation of the patient’s left and right hippocampal volumes warrants further consideration. Therefore, caution should be exercised regarding the constraints inherent in the measurement method. This meta-analysis aimed to determine the left and right hippocampal volume changes and the volume measurement method.

**Methods:**

The PubMed, Web of Science, and Scopus databases were searched and published until September 2023. A total of 29 studies were included in this meta-analysis.

**Results:**

The meta-analysis results indicate a significant reduction in total hippocampal volume in AD patients. The results for the hippocampal volume on the left and right sides of 3 classes, such as AD, mild cognitive impairment (MCI), and normal control (NC), showed a greater reduction on the left side. The results showed in AD: 95% confidence interval (CI) = −142 to 1.61, *P* = .04, *I*^2^ = 44%; MCI: 95% CI= −186 to −72, *P* = <.00001, *I*^2^ = 0%, and NC: 95% CI = −141 to 14, *P* = .11, *I*^2^ = 80%, respectively, with random effect. The left hippocampal in AD is smaller, while the right hippocampal is larger. The AD class significantly reduced hippocampal volume on both the left and right sides.

**Conclusion:**

Notably, automatic segmentation methods impact hippocampal volume measurements, with results potentially relying heavily on their correlation with the manual labeling process.

**Highlights**

[1] The total volume of Alzheimer disease (AD) classes had a smaller hippocampal than the mild cognitive impairment (MCI) and normal control (NC) classes.
[2] Volumetric asymmetry existed between the left and right hippocampal among the 3 classes (i.e., AD, MCI, and NC).
[3] The left hippocampal volume was reduced in the AD, MCI, and NC classes.
[4] The comparison of left and right hippocampal volume was found to be smaller in the MCI class.
[5] Automatic segmentation revealed a smaller left hippocampal, whereas the right hippocampal was larger on the right side.
[6] The automatic segmentation method for measuring hippocampal volume may rely highly on correlation with the manual labeling process.

## 1. Introduction

Alzheimer disease (AD) is the most prevalent neurodegenerative disease and is characterized by progressive memory loss and neuropsychiatric symptoms. AD is the most common cause of dementia, characterized by a progressive decline in cognitive function, and is estimated to cost the US healthcare system $172 billion per year.^[1]^ The hippocampal rapidly loses tissue, is functionally disconnected from other brain regions, and significantly affects its functional role in memory.^[2]^ Furthermore, previous studies found that there is a significant correlation between changes in hippocampal volume and the clinical symptoms of cognitive impairment in AD patients.^[3]^ Thus, the hippocampal is the most established structural biomarker for AD and is widely utilized to assess the progression of neurodegeneration in this condition.^[4–6]^

Hippocampal volume reduction is related to the disease progression. It is the most important indicator of cognitive function in patients with AD.^[7–10]^ Hippocampal volume is implied as a specific indicator of conversion from MCI to AD.^[11]^ Previous studies have indicated that reduced left and right hippocampal volumes can predict disease progression.^[12,13]^ For instance, several studies have reported varying results regarding hippocampal volume reductions. Some studies have identified a significant volume reduction in the left hippocampal,^[14,15]^ while other studies have found a significant volume reduction in the right hippocampal. In contrast, a previous study reported a significant volume reduction on the right side.^[9,16]^ These discrepancies highlight the need to investigate the differences between left and right hippocampal volume changes. Therefore, a detailed examination of the left and right hippocampal volumes is warranted to clarify these inconsistencies.

Furthermore, to elucidate this issue, it is imperative to consider the limitations inherent in the methods used to obtain the left and right hippocampal volumes. Specifically, delineating hippocampal techniques to quantify hippocampal volume is crucial for facilitating a comprehensive diagnosis of AD.^[4,17–19]^ The accurate segmentation of the hippocampal is an important step in studying hippocampal volume changes and related neurological disorders caused by the impairment of the hippocampal. There are several methodologies to assess hippocampal volume alterations in AD, such as manual and automated segmentation techniques to derive volumetric measures of the hippocampal.^[5,18,20,21]^ Manual segmentation is widely acknowledged as the gold standard for acquiring precise hippocampal volume measurements.^[21]^ However, manual segmentation is time-consuming, requires medical experts’ neuroanatomical knowledge, and is prone to inter-rater variability.^[22]^ Automated segmentation is popular in clinics for AD diagnosis nowadays.^[17]^ Although the automated method has shown good reproducibility and is comparable in accuracy to manual segmentation,^[23,24]^ a significant discrepancy between the automated and manual segmentation methods was found in the 5% to 35% range for the total hippocampal volume.^[25–27]^ The current body of literature indicates inconclusive findings regarding alterations in the left and right hippocampal volumes, accompanied by inherent complexities and potential sources of measurement uncertainty. To address this uncertainty, the automated and manual segmentation performances were compared to analyze the estimated volume^[6]^; it is necessary to ensure reliable hippocampal volume measurements.^[28]^ Therefore, determining the segmentation method is necessary because each method may approach distinct advantages, limitations, and appropriate use cases in medical imaging, computer vision, and remote sensing. Moreover, the findings offer valuable insights for appraising the accuracy of segmentation techniques for quantifying hippocampal volume.

We focused on the reduced volume changes in the left and right hippocampal by applying the segmentation volume measurement method. We further evaluated the automatic and manual segmentation methods by comparing studies on the left and right hippocampal volumes. Therefore, our hypothesis suggests the presence of discrepancies in the left and right hippocampal volumes when employing both automatic and manual segmentation methods. Moreover, these findings may yield valuable insights into accurately measuring hippocampal volume in the context of AD diagnosis. This meta-analysis aimed to determine the left and right hippocampal volume changes in AD using manual and automatic segmentation methods and to guide future research and clinical approaches for more accurate diagnoses and treatments. Likewise, it will enhance our understanding of the differentiation between automatic and manual segmentation methods for quantifying the hippocampal volume.

## 2. Methods

This systematic review and meta-analysis were performed according to the preferred reporting items for systematic reviews and meta-analyses guidelines (PRISMA).^[29]^ In addition, this research was registered with International Prospective Register of Systematic Reviews (PROSPERO), with the registration number CRD42024581493. Given that our study focused on systematic review and meta-analysis, there was no necessity for ethical review board approval or obtaining informed consent from the participants.

### 2.1. Eligibility criteria

Only studies with human participants are eligible for inclusion. Participants could be from any dataset, and there were no sex, age, or weight restrictions. However, for studies to meet inclusion criteria, the participants had to be shown the hippocampal volume in 3 classes (e.g., AD, MCI, and normal control [NC]) in magnetic resonance imaging (MRI) images. Consequently, the other regions with the shown volume were excluded, including the amygdala, corpus callosum, and other brain regions in MRI images. Included studies were required to have a method measurement of hippocampal volume; subjects: AD, MCI, and NC; the hippocampal region; and derived from structural MRI brain scans. Reviews, case reports, thesis papers, dissertations, conference abstracts, and letters were excluded. Only articles written in the English language were included. Exclusion criteria were exclusive focus on clinical/pathological populations; exclusion of the regions (e.g., amygdala); case studies, theses, book chapters, author responses, conference papers, posters, reviews, non-peer reviewed publications, published abstracts or any other reports without full text; non-English-language publications; and animal studies.

### 2.2. Search strategy

In this meta-analysis, we conducted a comprehensive literature search using 3 major databases: PubMed, Web of Science, and Scopus. The data extraction included all relevant studies published up to September 2023. This approach ensured an extensive and systematic collection of pertinent research articles to provide a robust basis for our analysis using the following keywords: Alzheimer OR Alzheimer AND Hippocampus OR Hippocampal AND Volume OR Brain Volume OR Volumetry AND segmentation. For detailed information, see Appendix 1, Supplemental Digital Content, http://links.lww.com/MD/O461. The search was limited to articles published in the English language. Two reviewers (YP and CWL) independently screened titles and abstracts and identified potentially relevant articles for full-text review. Subsequently, they independently conducted a full-text review and jointly decided on the final pool of articles included in the review. Reference list searches (i.e., backward reference searches) and cited reference searches (i.e., forward reference searches) were conducted on full-text articles that met the study selection criteria identified through the keyword search. Articles identified from the backward and forward reference searches were further screened and evaluated based on the same study selection criteria. All newly identified articles were analyzed until no additional relevant articles were found.

### 2.3. Data extraction

Two independent investigators (YP and CWL) systematically collected data, including publication date, author, study design, dataset characteristics, and MRI volume measurements. Data extraction focused on measurements of hippocampal volumes for both the left and right hemispheres and the total volume (combined left and right hippocampal volumes). Studies included in the analysis provided hippocampal volume data for 3 diagnostic categories: Alzheimer disease (AD), mild cognitive impairment (MCI), and NC. Hippocampal volume measurements derived from manual and automatic segmentation methods were recorded separately to evaluate the potential impact of the segmentation technique on volumetric outcomes. Variance data from the included studies were transformed into mean values and standard deviations (SDs) to standardize comparisons. Our analysis primarily examined differences in left and right hippocampal volumes across the 3 diagnostic groups, with additional exploration of the effects of segmentation methodology on hippocampal volume estimates.

### 2.4. Meta-analysis

Data analyses were performed using the standardized mean difference with 95% confidence intervals (CIs) to quantify the mean differences in left and right hippocampal volumes across the 3 groups (i.e., AD, MCI, and NC). Statistical heterogeneity was evaluated using the *I*^2^ index.^[30]^ The level of heterogeneity represented by the *I*^2^ index was interpreted as small (*I*^2^ ≤ 25%), moderate (25% < *I*^2^ ≤ 50%), large (50% < *I*^2^ ≤ 75%), or very large (*I*^2^ > 75%). The pooled sensitivity and specificity values with 95% CI were calculated using bivariate random-effects modeling.^[31,32]^ Since heterogeneity might indicate subgroup effects, we also explored heterogeneity in the pooled results using subgroup analysis. Subgroup analyses were conducted in terms of the 3 classes (e.g., AD, MCI, and NC) of the left hippocampal, the right hippocampal, the total volume of hippocampal, and the segmentation method used in the study. A fixed-effects model is warranted in cases characterized by modest or limited heterogeneity. Contrastingly, a random-effects model is deemed appropriate for estimating substantial or exceedingly large heterogeneity. Meta-analysis was performed using Review Manager (version 5.3; The Nordic Cochrane Center, The Cochrane Collaboration). Meta-analysis was performed using Review Manager (version 5.3; The Nordic Cochrane Center, The Cochrane Collaboration).

### 2.5. Quality assessment of the studies

The randomized controlled trials (RCTs) quality was assessed using a 6-item bias assessment scale. This scale systematically evaluates potential sources of bias, ensuring a rigorous appraisal of study quality across key domains,^[33]^ including random sequence, allocation concealment, blinding, incomplete outcome data, selective reporting, and other biases.

## 3. Results

### 3.1. Study selection

A comprehensive search across 3 databases yielded 4795 studies using the specified retrieval strategy. After removing 210 duplicate articles, 4585 unique studies remained. These studies underwent an initial screening based on titles, keywords, and abstracts, identifying 254 studies as potentially relevant to our research question.

**Figure 1.**
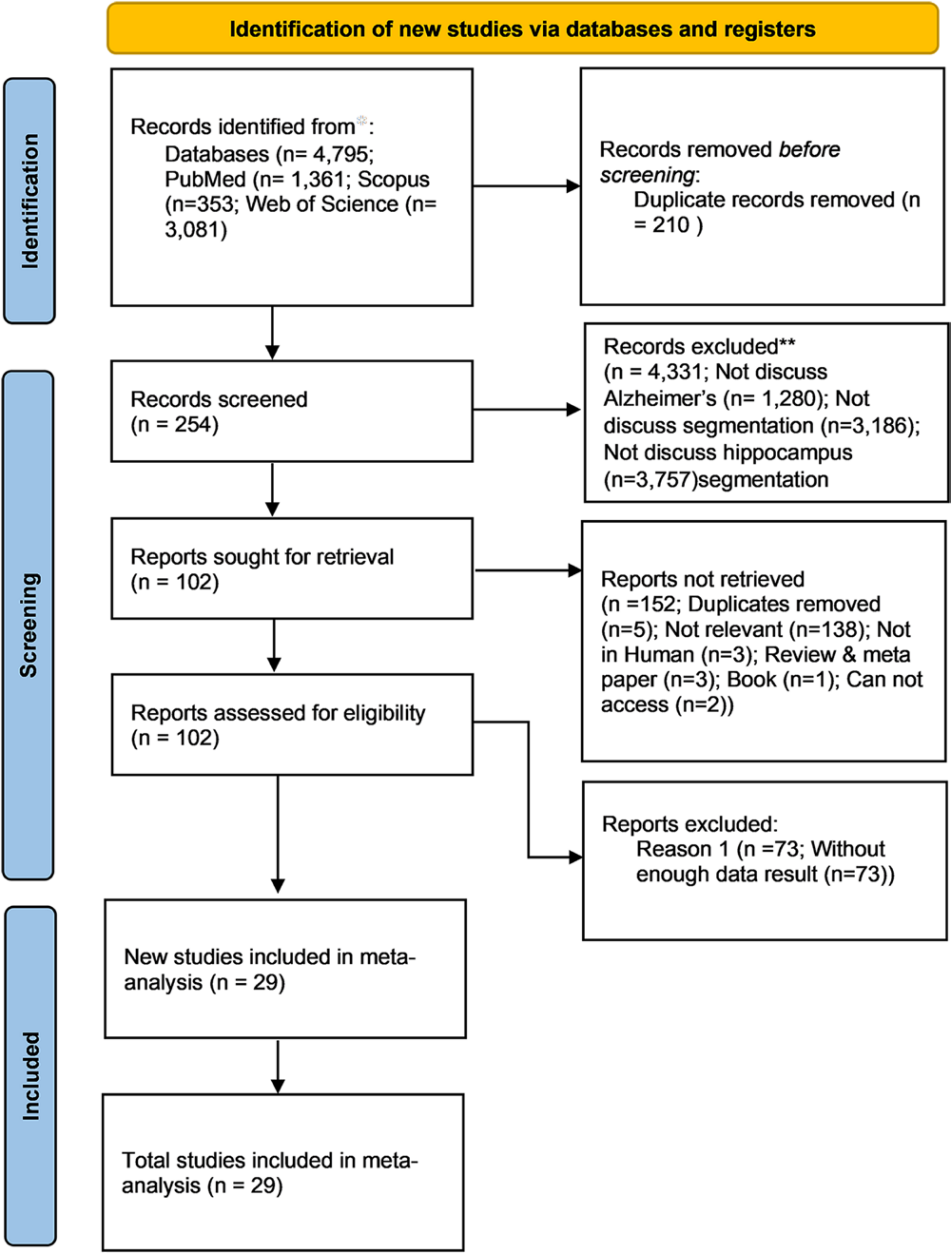
Literature screening flow chart.

For a more thorough assessment, we retrieved the full texts of these 254 studies. During this review process, we excluded an additional 220 studies for the following reasons: 5 were duplicates, 138 lacked sufficient data for analysis, 3 were conducted on non-human subjects, 3 were systematic reviews rather than original research articles, 1 was a book, and 73 were determined to be irrelevant to the topic. Ultimately, 29 studies met all inclusion criteria and were included in the meta-analysis. These studies provided data essential for our investigation and aligned with our predetermined criteria, ensuring the robustness and relevance of our analysis.^[4,5,18–21,34–55]^ The literature screening process is illustrated in Figure 1.

### 3.2. Characteristics of selected studies

The basic characteristics of the included studies are summarized in Table 1. We have provided a summary of the author, cohort name, total sample size for each class, and the hippocampal volume for both the left and right hippocampal among 3 classes (e.g., AD, MCI, and NC). The left and right hippocampal volumes in AD, MCI, and NC were summarized; fourteen studies were in AD.^[5,18,20,21,36,46–54]^ In the AD class, 4 studies used manual segmentation to obtain the left and right hippocampal volumes.^[5,21,52,53]^ While 8 studies used the automatic segmentation method,^[18,20,46,47,49,51,54]^ 2 other studies used both automatic and manual,^[48,50]^ and 1 used the semi-automatic method.^[36]^ Ten studies were in MCI.^[5,18,20,21,36,47,48,50,54,55]^ In the MCI class, 3 studies used the manual segmentation method.^[5,21,48]^ At the same time, 5 studies used the automatic method,^[18,20,47,54,55]^ and 2 other studies used both automatic and manual.^[48,50]^ One used the semi-automatic method.^[36]^ Fourteen studies were in NC.^[5,18,20,21,36,45–50,52,53,55]^ In the NC class, 4 studies used manual methods.^[5,21,52,53]^ Seven studies used the automatic segmentation method,^[18,20,46–49,55,56]^ 2 other studies used both automatic and manual.^[48,50]^ One used the semi-automatic method.^[36]^ In the end, 2 studies were utilized to differentiate between the automatic and manual segmentation methods for measuring hippocampal volumes^.[48,50]^

**Table 1:** The characteristics of selected studies.

No	Author	Cohort name	Segmentation	Sample size			Hippocampal volume mean (SD) mm^3^								
				n (AD)	n (MCI)	n (NC)									
							Left	Right	Total	Left	Right	Total	Left	Right	Total
							AD			MCI			NC		
1	Boccardi et al, 2015^[18]^	ADNI	Manual	23	23	31	1788 (342)	1820 (369)	N/A	1957 (348)	2029 (372)	N/A	2443 (291)	2429 (303)	N/A
2	Boccardi et al, 2 2015^[18]^	ADNI 1.5T	HarP	22	22	22	2405 (507)	2487 (543)	N/A	2620 (447)	2647 (506)	N/A	3119 (533)	3156 (303)	N/A
		ADNI 3T	HarP	23	23	22	2203 (467)	2405 (516)	N/A	2569 (456)	2729 (441)	N/A	3001 (452)	3084 (303)	N/A
3	Cherubini et al, 2010^[46]^	N/A	Automatic	30	30	30	3952 (582)	4159 (561)	N/A	4395 (559)	4601 (545)	N/A	4806 (464)	5038 (303)	N/A
4	Dhikav et al, 2016^[5]^	dept.Neurology	Manual	20	13	32	1590 (550)	1640 (550)	N/A	2360 (380)	2350 (420)	N/A	2770 (600)	2730 (303)	N/A
5	Du et al, 2001^[36]^	N/A	semi-automatic	29	36	40	2231 (506)	2364 (545)	4595 (1009)	2783 (511)	2874 (401)	5657 (864)	3135 (403)	3191 (303)	6327 (799)
6	Liu et al, 2020^[47]^	ADNI	Deep CNN	97	97	119	3203 (592)	3310 (674)	N/A	3471 (562)	3608 (574)	N/A	4139 (524)	4221 (303)	N/A
7	Sánchez-Benavides et al, 2010^[48]^	Hospital clinic and del mar	Manual	25	23	41	2494 (494)	2587 (515)	N/A	2835 (465)	2949 (546)	N/A	3342 (414)	3406 (303)	N/A
			Freesurfer	25	23	41	2632 (562)	2684 (565)	N/A	3050 (490)	3099 (540)	N/A	3452 (575)	3628 (303)	N/A
8	Shen et al, 2012^[49]^	MDMSMC	MNI	46	46	103	1520 (330)	1430 (390)	N/A	1753 (260)	1577 (320)	N/A	1995 (260)	1834 (303)	N/A
			MNI-S	46	46	103	2056 (420)	1916 (450)	N/A	2308 (280)	2146 (380)	N/A	2602 (240)	2464 (303)	N/A
		MDMSMC	MNI-E	46	46	103	2291 (470)	2153 (500)	N/A	2621 (330)	2411 (370)	N/A	3025 (290)	2828 (303)	N/A
		MDMSMC	Neuroquant	25	25	21	1866 (340)	1911 (460)	N/A	2139 (240)	2163 (310)	N/A	2388 (260)	2537 (303)	N/A
9	Wolf et al, 2017^[50]^	ADNI	Automatic	45	45	44	2179 (436)	2635 (466)	N/A	2570 (401)	2933 (423)	N/A	2877 (393)	3255 (303)	N/A
		ADNI	Manual	45	45	44	2339 (497)	2486 (548)	N/A	2644 (463)	2732 (479)	N/A	3108 (532)	3185 (303)	N/A
10	Lee et al, 2022^[51]^	PNUH	Freesurfer	109	109		2470 (450)	2540 (450)	N/A	N/A	N/A	N/A	N/A	N/A	N/A
11	Lim et al, 2013^[52]^	NINCD	Manual	31		33	3068 (525)	3077 (609)	N/A	N/A	N/A	N/A	3818 (441)	3984 (303)	N/A
12	Boccardi et al, 2011^[21]^	OutpatienMemory Clinic	Manual	20	20	19	3609 (827)	3645 (799)	N/A	N/A	N/A	N/A	4914 (745)	5070 (303)	N/A
13	Yoon et al, 2019^[54]^	Duke Memory Disorders Clinic.	Automatic	9	9	N/A	3370 (660)	3630 (530)	N/A	3130 (420)	3300 (530)	N/A	N/A	N/A	N/A
14	Eckerström et al, 2011^[55]^	N/A	Freesurfer	44	44	92	3800 (400)	3900 (500)	N/A	N/A	N/A	N/A	3900 (400)	4000 (303)	N/A
15	Li et al, 2016^[56]^	N/A	Freesurfer	N/A	N/A	26	N/A	N/A	N/A	N/A	N/A	N/A	3300 (407)	3428 (303)	N/A
16	Shi et al, 2009^[57]^	EADC-ADNI	Manual	45	45	45	N/A	N/A	4831 (1003)	N/A	N/A	5337 (878)	N/A	N/A	6291 (973)
		EADC-ADNI	AccuBrain	45	45	45	N/A	N/A	5409 (948)	N/A	N/A	5872 (907)	N/A	N/A	6632 (811)
		EADC-ADNI	FreeSurfer	45	45	45	N/A	N/A	6214 (1180)	N/A	N/A	6899 (1051)	N/A	N/A	7961 (1257)
17	Apostolova et al, 2015^[4]^	ADNI	HarP	9		7	N/A	N/A	1867 (532)	N/A	N/A		N/A	N/A	2837 (427)
18	Bhagwat et al, 2016^[35]^	ADNI	Majority VOTE	20	20	20	N/A	N/A	1897 (582)	N/A	N/A	1960 (599)	N/A	N/A	2084 (615)
		ADNI	STAPLE	20	20	20	N/A	N/A	2068 (655)	N/A	N/A	2124 (649)	N/A	N/A	2236 (659)
		ADNI	JLF	20	20	20	N/A	N/A	1697 (551)	N/A	N/A	1803 (572)	N/A	N/A	1943 (593)
		ADNI	AWoL-MRF	20	20	20	N/A	N/A	2047 (631)	N/A	N/A	2147 (652)	N/A	N/A	2312 (676)
19	Colliot et al, 2008^[19]^	CHU	Automated	17	17	17	N/A	N/A	1950 (460)	N/A	N/A	2300 (460)	N/A	N/A	2860 (460)
20	Firbank et al, 2008^[37]^		Manual	9		9	N/A	N/A	1615 (546)	N/A	N/A		N/A	N/A	2884 (368)
			Automated	9	9	9	N/A	N/A	1869 (530)	N/A	N/A		N/A	N/A	2769 (331)
21	Ingala et al, 2022^[38]^	ADC	FSL	109	209	209	N/A	N/A	2370 (550)	N/A	N/A	2700 (570)	N/A	N/A	2970 (540)
		LMC	FSL	96	70	70	N/A	N/A	2170 (510)	N/A	N/A	2560 (630)	N/A	N/A	2660 (620)
		ADC	Freesurfer	109	209	209	N/A	N/A	3330 (470)	N/A	N/A	3600 (490)	N/A	N/A	3910 (390)
		LMC	Freesurfer	96	70	70	N/A	N/A	3150 (490)	N/A	N/A	3320 (520)	N/A	N/A	3740 (620)
22	Cardoso et al, 2013^[39]^	ADNI	Manual	147	335	200	N/A	N/A	4218 (903)	N/A	N/A	4733 (781)	N/A	N/A	5152 (656)
23	Lee et al, 2017^[40]^	KUDMC	FMRIBs	107		37	N/A	N/A	8300 (1400)	N/A	N/A		N/A	N/A	9300 (1200)
24	Leung et al, 2010^[41]^	ADNI	Automated	147	335	200	N/A	N/A	3984 (784)	N/A	N/A	4450 (766)	N/A	N/A	5251 (659)
25	Platero and Tobar, 2017^[42]^	ADNI	FS	45	45	45	N/A	N/A	3510 (820)	N/A	N/A	4060 (750)	N/A	N/A	4780 (580)
		ADNI	MV	45	45	45	N/A	N/A	3150 (500)	N/A	N/A	3420 (490)	N/A	N/A	3870 (350)
		ADNI	Pb	45	45	45	N/A	N/A	3080 (520)	N/A	N/A	3410 (580)	N/A	N/A	3910 (400)
		ADNI	DS	45	45	45	N/A	N/A	3290 (450)	N/A	N/A	3580 (480)	N/A	N/A	4010 (320)
		ADNI	PT	45	45	45	N/A	N/A	3230 (510)	N/A	N/A	3560 (530)	N/A	N/A	4040 (370)
		ADNI	GT	45	45	45	N/A	N/A	3140 (660)	N/A	N/A	3530 (630)	N/A	N/A	4190 (550)
		LCCN	FS	13	97	52	N/A	N/A	3880 (820)	N/A	N/A	4440 (750)	N/A	N/A	5030 (580)
		LCCN	MV	13	97	52	N/A	N/A	3080 (250)	N/A	N/A	3320 (370)	N/A	N/A	3600 (280)
		LCCN	Pb	13	97	52	N/A	N/A	3320 (370)	N/A	N/A	3410 (510)	N/A	N/A	3910 (370)
		LCCN	DS	13	97	52	N/A	N/A	3390 (450)	N/A	N/A	3700 (480)	N/A	N/A	4040 (320)
		LCCN	PT	13	97	52	N/A	N/A	3370 (510)	N/A	N/A	3710 (530)	N/A	N/A	4070 (370)
26	Su et al, 2018^[43]^	Cambridgeshire	Manual	12	20	25	N/A	N/A	2400 (400)	N/A	N/A	2500 (800)	N/A	N/A	3300 (300)
27	Wolz et al, 2014^[44]^	ADNI 1.5 T	LEAP	28	74	51	N/A	N/A	1964 (340)	N/A	N/A	2162 (340)	N/A	N/A	2447 (270)
		ADNI 3T	LEAP	28	74	51	N/A	N/A	1992 (334)	N/A	N/A	2192 (334)	N/A	N/A	2461 (292)
28	Yi et al, 2016^[45]^	ADC	FMRIBs	391	201	181	N/A	N/A	8400 (1300)	N/A	N/A	8900 (1300)	N/A	N/A	9800 (1200)
29	Qu et al, 2023^[20]^	department of neurology	Automatic	21	39	32	2733 (461)	2805 (478)	N/A	3260 (805)	3279 (339)	N/A	3449 (294)	3636 (302)	N/A

Abbreviations: AD = Alzheimer disease, ADNI = Alzheimer disease neuroimaging initiative, AWoL-MRF = autocorrecting walks over localized Markov random field model, CNN = convolutional neural network, DS = non-rigid registrations, EADC-ADNI = European Alzheimer Disease Consortium – Alzheimer Disease Neuroimaging Initiative, FMRIBs = functional magnetic resonance imaging of the brain, FSL = FMRIB Software Library, HaRP = hippocampal subfield and resting-state fMRI pipeline, JLF = joint label fusion, LCCN = law enforcement action partnership, LEAP = Learning Embeddings for Atlas Propagation, MCI = mild cognitive impairment, MNI = Montreal Neurological Institute, MV = majority voting, N/A = not available, NC = normal control, n = number of subjects, Pb = patch-based, PT = point tracker, STAPLE = simultaneous truth and performance level estimation.

### 3.3. Study quality assessment

**Figure 2.**
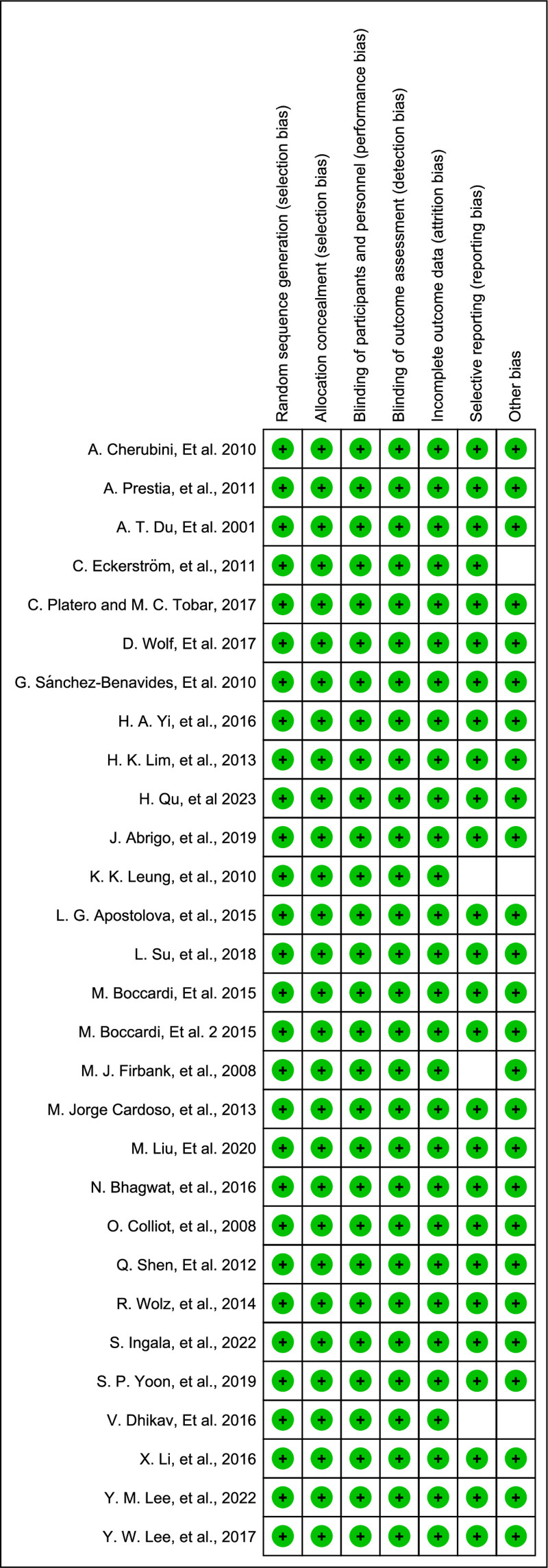
Risk-of-bias summary of each included study.

**Figure 3.**
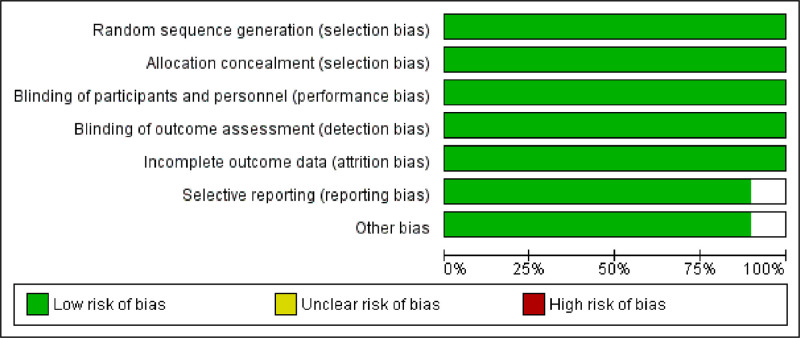
Risk-of-bias graph: review the author’s judgments about each risk-of-bias item presented as percentages across all included studies.

The study quality assessments provided information regarding the bias of RCTs. The Cochrane Handbook risk-of-bias assessment tool was used to assess the methodological quality of RCTs. Overall, the results showed low risk (Figs. 2 and 3).

### 3.4. Hippocampal total volume In AD, MCI, and NC

**Figure 4.**
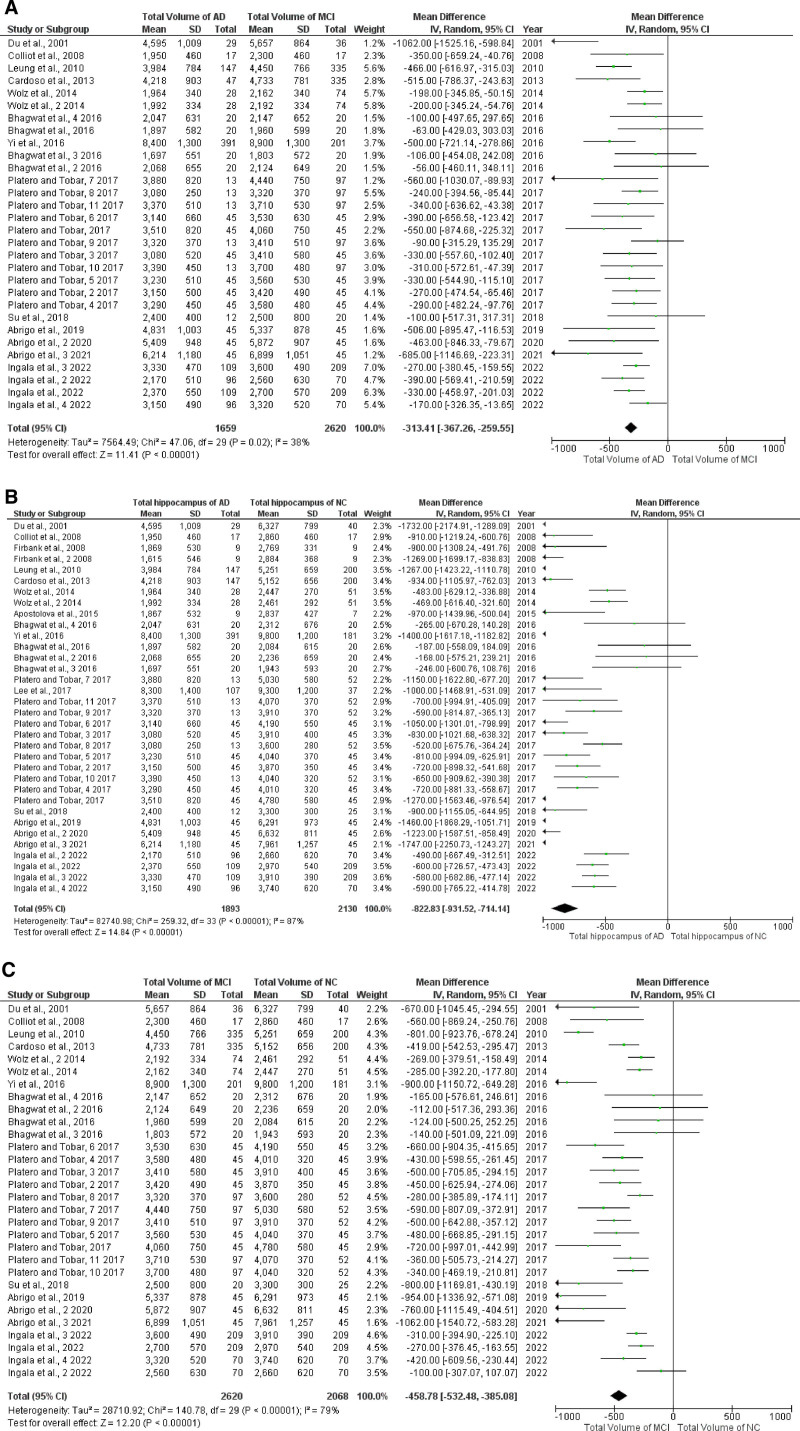
Forest plot of left hippocampal volume; (A) AD vs MCI; (B) AD vs NC; and (C) MCI vs NC. AD = Alzheimer disease, MCI = mild cognitive impairment, NC = normal control.

A comparison of the total hippocampal volume (summary of left and right) was made; there were 11 studies in AD versus MCI and MCI versus NC^[19,34–36,38,39,41–45]^;14 studies were in AD versus NC^.[4,19,34–45]^ The meta-analysis results showed that the hippocampal volume was reduced to a greater extent in patients with AD (Fig. 4). The *Q*-test for heterogeneity indicated that the comparison between AD and NC was not significant (*P* = .02). In contrast, the comparisons between MCI and NC (*P* < .00001), as well as between AD and MCI (*P* < .00001), demonstrated a high level of significance. These results suggest that significant heterogeneity exists in the comparisons involving MCI and NC and AD and MCI, warranting further investigation into the underlying factors contributing to these differences.

### 3.5. Hippocampal left and right volume in AD, MCI, and NC

**Figure 5.**
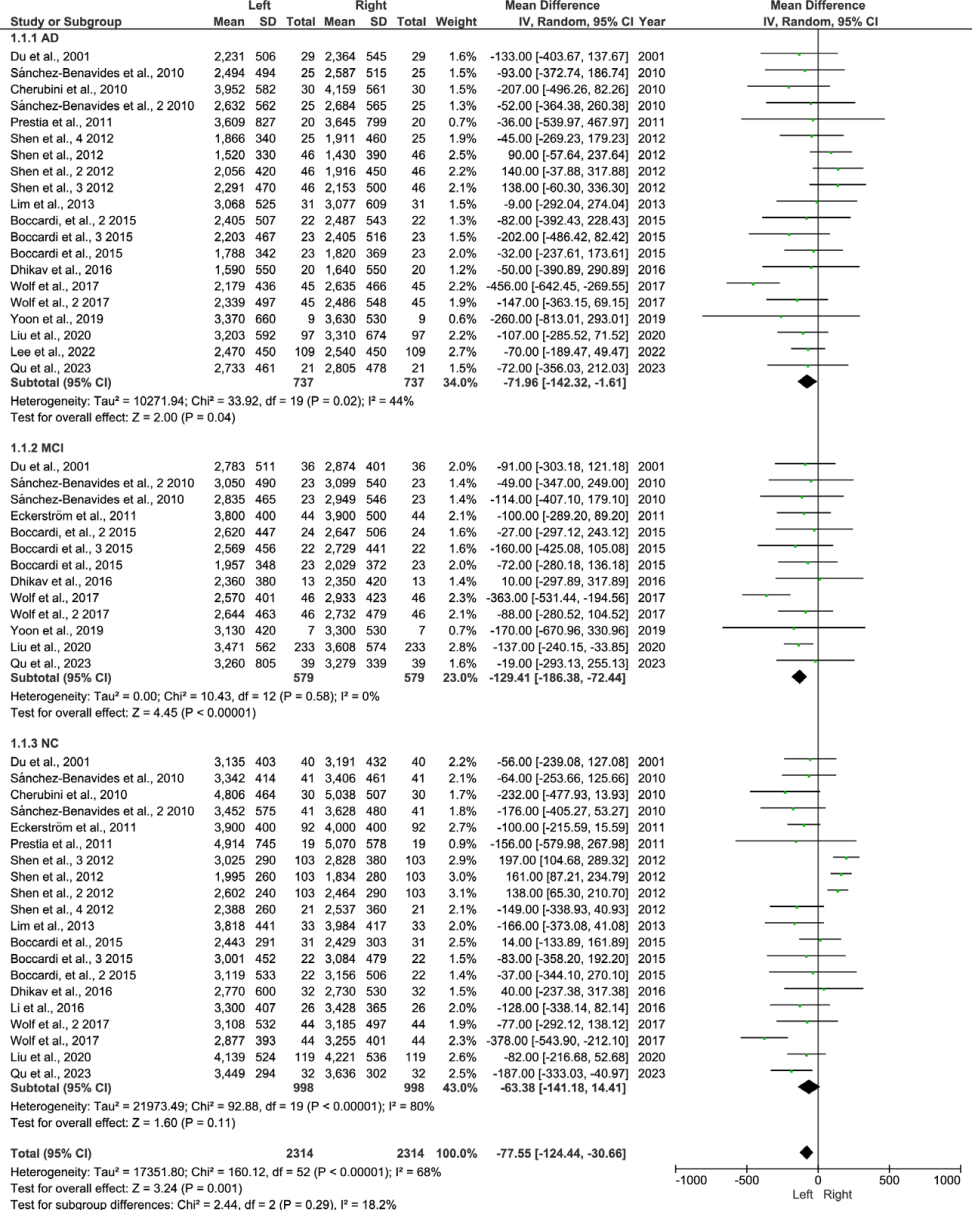
Forest plot of hippocampal volume left and right in AD, MCI, and NC. AD = Alzheimer disease, CI = confidence interval, MCI = mild cognitive impairment, NC = normal control, SD = standard deviation.

A total of 15 studies^[5,18,20,21,36,45–55]^ assessed the left and right hippocampal volumes in AD, MCI, and NC. As shown in Figure 5, the results for the hippocampal volume on the left and right sides showed a greater reduction on the left side. The results showed in AD: 95% CI= −142 to 1.61, *P* = .04, *I*^2^ = 44%, random effect; MCI: 95% CI = −186 to −72, *P* = <.00001, *I*^2^ = 0%, random effect, and NC: 95% CI = −141 to 14, *P* = .11, *I*^2^ = 80%, random effect, respectively.

### 3.6. Comparison of left and right hippocampal volume change

**Figure 6.**
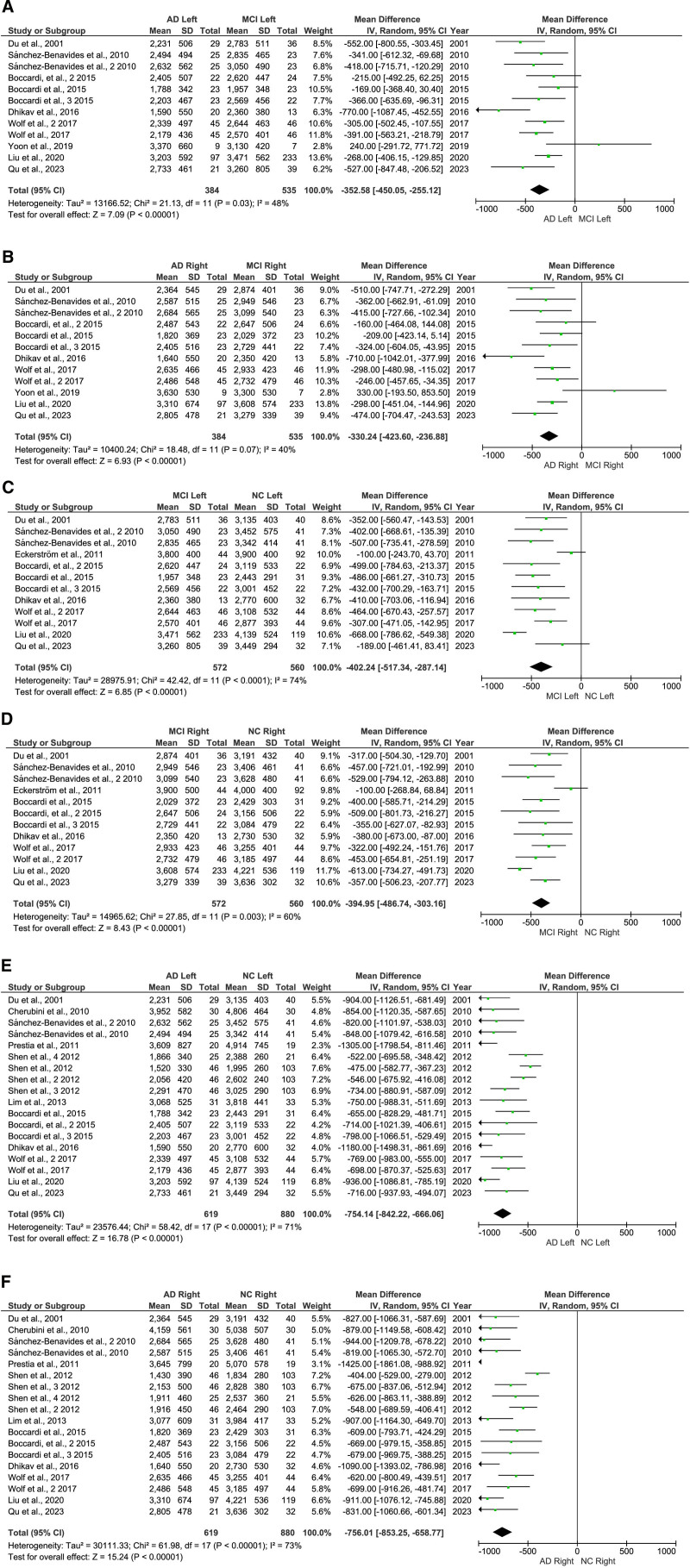
Forest plot of left and right hippocampal volume; **(A**) AD versus MCI left; **(B**) AD versus MCI right **(C**) MCI versus NC left; **(D**) MCI versus NC right; **(E**) AD versus NC left; **(F**) AD versus NC right. AD = Alzheimer disease, CI = confidence interval, MCI = mild cognitive impairment, NC = normal control, SD = standard deviation.

We also investigated the volume changes between the left and right hippocampal in the AD, MCI, and NC groups. There were 9 studies of AD versus MCI^[5,18,20,21,36,47,48,50,54]^ Nine studies of MCI versus NC^[5,18,20,21,36,47,48,50,55]^; There were 13 studies of AD versus NC.^[5,18,20,21,36,46–50,52,53]^ The meta-analysis results showed that AD hippocampal volume was reduced to a greater extent in the left and right hippocampal (Fig. 6). Q-test of heterogeneity in the left hippocampal volume of AD versus MCI (*P* = .03), MCI versus NC (*P* < .00001), and AD versus NC (*P* < .00001). Simultaneously, the Q test of heterogeneity in the right hippocampal volume of AD versus MCI (*P* = .07), MCI versus NC (*P* = .003), and AD versus NC (*P* ≤ .00001).

### 3.7. Comparison of the segmentation methods

**Figure 7.**
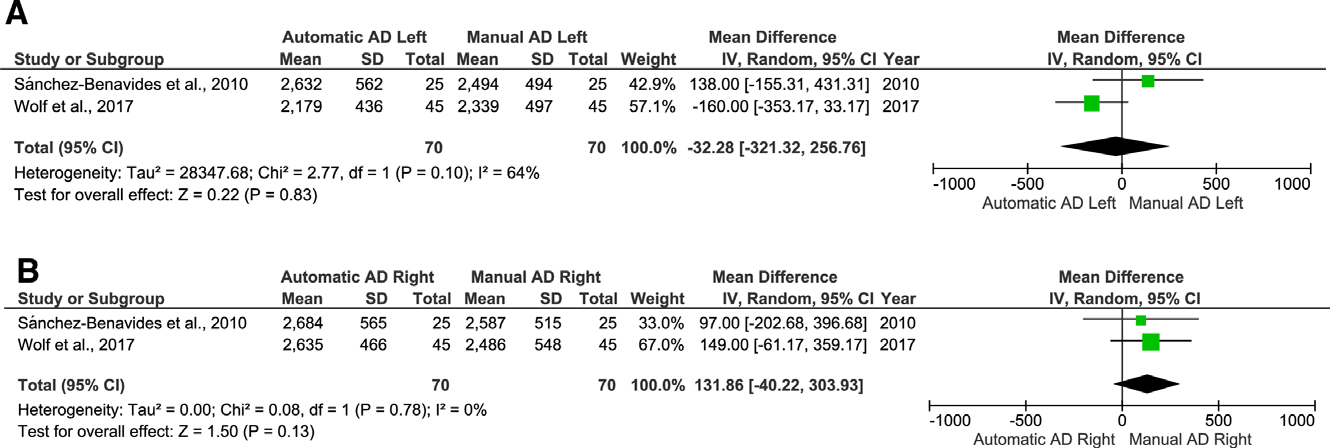
Forest plot of automatic and manual segmentation method in AD; **(A**) The left hippocampal; **(B**) The right hippocampal. AD = Alzheimer disease, CI = confidence interval, SD = standard deviation.

We also investigated the effect of the segmentation method, including 2 studies^[48,50]^ on automatic and manual segmentation in the left hippocampal of AD patients, which were included in the meta-analysis. The results indicated that the AD class’s automatic segmentation of the left hippocampal region was smaller than the right hippocampal region, which exhibited larger automatic segmentation (Fig. 7). Notably, the automatic and manual segmentation methods for the left hippocampal demonstrated an *I*^2^ value of 64%, suggesting substantial heterogeneity. In contrast, the right hippocampal showed an *I*^2^ value of 0%, indicating no observed heterogeneity in this region.

## 4. Discussion

This study found that the hippocampal volume changes in the left and right sides were reduced among the 3 classes (i.e., AD, MCI, and NC). In addition, we investigated the impact of the volume reduction in the left hippocampal in the AD, MCI, and NC classes. Regarding this hypothesis, we found that automatic segmentation on the left side was larger than on the right. Thus, we may say that the segmentation method could affect the results of hippocampal volume changes on the left and right sides.

The total hippocampal volume (sum of the right and left sides) was also the smallest in the AD class. We also found a separate comparison between the left and right hippocampal volumes in the 3 classes (i.e., AD, MCI, and NC); both sides were reduced to a greater extent in the AD classes. This result was similar to those described in previous studies; total hippocampal volume decreased in patients with AD compared to those with MCI and NC.^[13,58]^ Several factors contribute to smaller hippocampal volume in AD, such as plasma Apolipoprotein E (ApoE) levels as a genetic risk factor,^[59]^ higher thyroid function, and serum thyroid hormone levels^.[60]^ In addition, serum cortisol levels are associated with smaller hippocampal volumes,^[61]^ and to prevent hippocampal volume loss in older adults, they need to maintain mental health.^[62]^ A close relationship between hippocampal volume and cognitive performance has been observed in AD.^[13]^ Because damage initially appears in the hippocampal, a part of the brain is essential for memory formation in AD^[63]^ and has been proposed as a possible surrogate biomarker.^[48]^ In addition, the human brain is typically characterized by bilateral symmetry.^[64]^ Notably, in the AD class, both the left and right hippocamp exhibit notable reductions in volume, leading to a discernible asymmetry in brain function. Thus, many factors may contribute to the development of AD and its impact on hippocampal volume. The hippocampal plays an important role in the diagnosis of AD and the development of possible treatments.

Furthermore, we found that the hippocampal volume was asymmetrical. Among the 3 classes (i.e., AD, MCI, and NC)., the left hippocampal volume was smaller than the right. Our findings were similar to those of a previous meta-analysis, in which the left hippocampal volume was smaller than that of the right.^[57]^ This is due to the higher atrophy of the contralateral left hippocampal volume, and this difference that this asymmetry contributes to cognitive deficits.^[5,15,56,57]^ Hence, a discernible asymmetry in the volumetric reduction between the left and right hippocampal is evident. Notably, the volumetric reduction observed in the left hippocampal is the most salient predictor of AD.^[65]^ We may assume that further investigation should prioritize the left hippocampal volume as a valuable tool for diagnosing AD.

**Figure 8.**
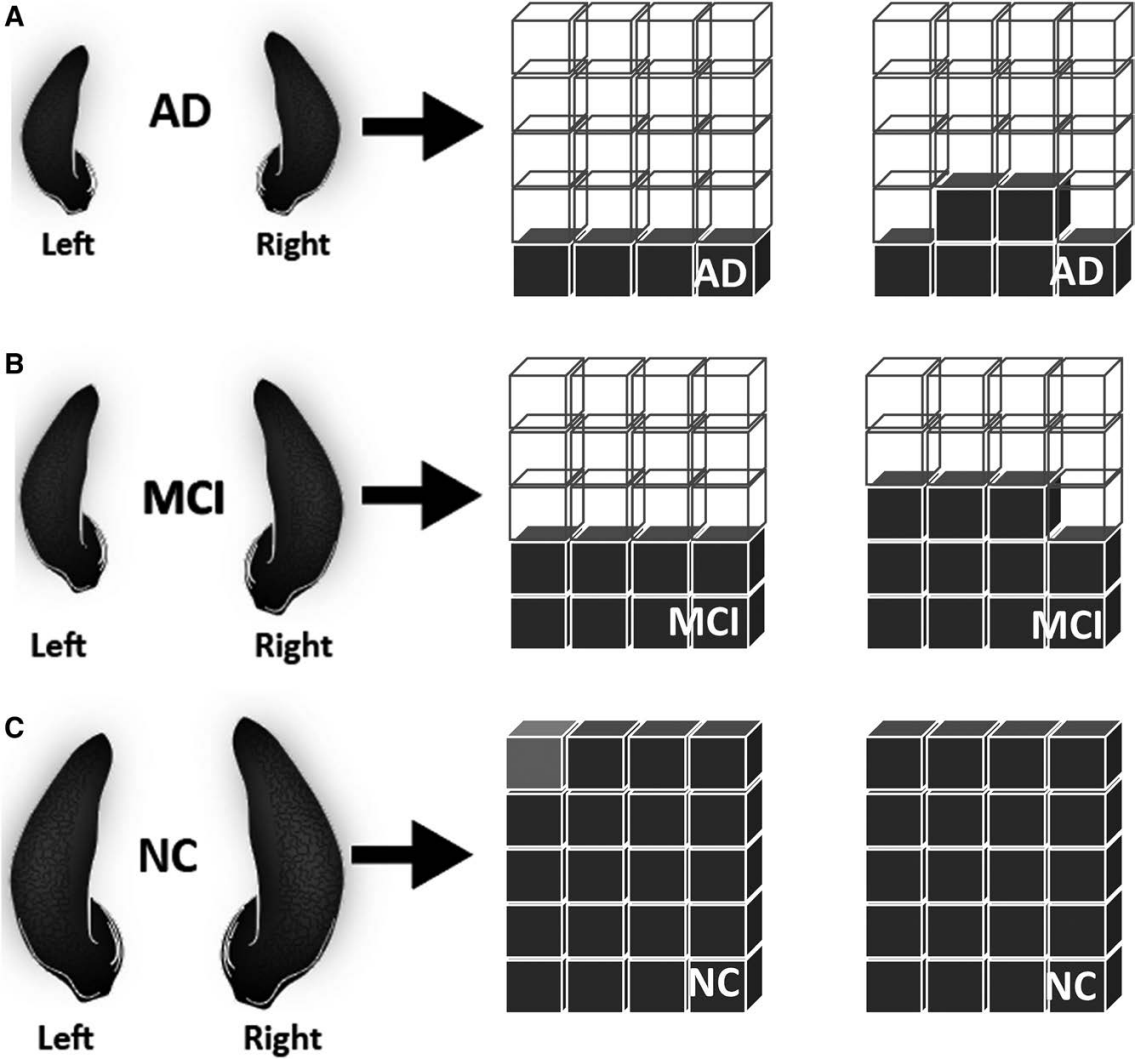
The illustration of the left and right reduction of the hippocampal volume in 3 classes (i.e., AD, MCI, and NC): (A) The left and right hippocampal volume reduced more on both sides; (B) The comparison of left and right hippocampal volume in MCI reduced more; and (C) The left and right hippocampal volume in NC class. AD = Alzheimer disease, L = Left hippocampal volume, MCI = mild cognitive impairment, NC = normal control, R = right hippocampal volume, SD = standard deviation.

In contrast, this study found that the comparison of the left and right hippocampal volumes was the lowest in MCI. This causes MCI to convert to dementia subsequently, and converters have an enhanced rate of left hippocampal volume loss.^[65]^ In addition, individuals harboring the ApoE4 allele may exhibit increased susceptibility to left hippocampal atrophy in MCI, with specific hippocampal subfields showing more pronounced involvement.^[66]^ This condition was significantly associated with conversions from MCI to AD,^[11]^ such as prodromal stages of MCI (i.e., early MCI and late MCI)^[67]^ and subtypes of MCI, to investigate the prognosis for AD development.^[68]^ Thus, from these findings, we could recognize the prodromal stages in earlier stages, and subtypes of MCI help characterize the diversity of cognitive decline and may help identify patients at high risk for progression to AD or dementia. However, it is possible to establish more specific therapies for early prevention. An illustration of the reduction in the hippocampal volume is shown in Figure 8.

The results showed that the automatic segmentation in the left hippocampal volume was considerably lower than in the right. Similar to the results of Wenger et al, the automatic segmentation of the left hippocampal was smaller than the manual segmentation in young adults.^[6]^ As illustrated in Figure 7, the automatic segmentation method indicated a larger volume for the left hippocampal than the right. In contrast, the manual segmentation method revealed larger volumes for both the left and right hippocampal when compared to the automatic results. This discrepancy is likely attributed to poor-quality images and significant degeneration of the left hippocampal in patients with epilepsy, which may have compromised the accuracy of the automatic segmentation results for the left hippocampal. Consequently, this suggests that manual segmentation may provide more reliable measurements in cases where image quality is suboptimal or structural abnormalities are present.^[69]^ In addition, owing to the imposition of an excessively high probability threshold, this outcome may have arisen conceivably because of the substantial dissimilarity of the subject from the training set.^[70]^ In contrast, previous studies found that automated segmentation predominantly highlights the right side.^[71,72]^ Even though manuals are considered the gold standard for accurately defining regions of interest like the hippocampal,^[21]^ we may consider the protocol to obtain the volume since it could make nuanced decisions. Whereas the automatic segmentation method can be more efficient and reduce bias, we may consider the possibility of larger area segmentation that will affect the volumes. In summary, the choice between automatic and manual segmentation methods should be guided by the study’s specific context, including the quality of the images, the complexity of the structures being analyzed, and the available resources. Understanding the strengths and limitations of each approach allows researchers and clinicians to make informed decisions that optimize the accuracy and efficiency of their analyses. In many cases, a hybrid approach that leverages both methods may provide the best results, combining the speed and consistency of automatic segmentation with the precision and flexibility of manual segmentation.

For this reason, it may cause an effect of sex differences, such as considering sex in assessing hippocampal volume changes, since mixed groups masked the extent of volume alterations.^[71]^ Additionally, the different patterns of change in females and males may be reflected in the different symptoms between sexes, and the asymmetric damage found in females may contribute significantly to sex-based differences. Thus, we may say that automated segmentation techniques may face challenges in delineating hippocampal structures under severe conditions such as AD. Hence, it is conceivable that measurement of hippocampal volume presents intriguing aspects when employing automated segmentation methods. Still, the variation between methods is noteworthy since it draws attention to the importance of the procedure in the dataset-processing step and the robustness of the brain anatomy. Further investigations are warranted to explore this subject. Nevertheless, it is pertinent to acknowledge that the limited scope of the studies in the comparison may potentially influence the outcomes, warranting further in-depth analysis.

A previously published meta-analysis concluded that there is a consistent pattern of left-less-than-right-asymmetric volume using manual segmentation.^[48,57]^ Simultaneously, the segmentation method is noteworthy because it draws attention to the importance of volume measurements. Thus, it is well known that the AD class generally exhibits smaller hippocampal volumes, and our contribution to conducting a systematic and rigorous approach is to quantify and understand the extent of this difference, account for potential influencing factors, and provide valuable insights for both research and clinical practice.

However, this meta-analysis has some limitations. First, we limited the search to MRI images because they are widely used to measure the hippocampal volume in AD. Previous studies have used CT to increase the accuracy of diagnosing AD. They found that the segmentation of cortical areas is typically involved in early AD on FDG PET/CT brain images and radiomics analysis to identify specific high-order features.^[73]^ PET/CT images may be used in further studies to determine changes in hippocampal volume. Second, our study used the hippocampal volumes on the left and right sides of patients with AD. Previous meta-analyses have used the volume of the olfactory cortex region in AD.^[74]^ Further studies may use other brain regions for meta-analysis. For instance, the left and right amygdala volume in AD is due to previous studies that only used the left and right amygdala in normal subjects or other regions as biomarkers in diagnosing AD.^[12,75]^ Ultimately, the comparison of segmentation methods in our analysis is limited due to the inclusion of only 2 studies, categorizing it as a small-scale investigation. Future research should explore additional parameters and factors that could influence the choice of method for measuring hippocampal volume. A broader range of studies and variables would enhance the understanding of the effectiveness and accuracy of both automatic and manual segmentation techniques.

## 5. Conclusion

In conclusion, this meta-analysis demonstrates a significant reduction in total hippocampal volume (the sum of left and right volumes) in individuals diagnosed with AD. Our findings reveal notable volumetric asymmetry between the left and right hippocampi across the 3 classes: AD, MCI, and NC. Specifically, the left hippocampal exhibited a more pronounced reduction in volume compared to the right hippocampal among these classes. Furthermore, the MCI class displayed smaller hippocampal volumes than those observed in both the left and right hippocampal of the NC class. The observed reductions in hippocampal volume may serve as critical biomarkers for early identification of AD, providing medical experts or professionals with valuable tools for diagnosis. Furthermore, understanding the volumetric asymmetry could inform targeted therapeutic strategies to mitigate cognitive decline associated with AD. The use of automated segmentation methods highlighted that the left hippocampal volume was smaller than that of the right hippocampal volume, which was comparatively larger. Our contribution lies in establishing a comparative analysis of left and right hippocampal volumes through both automatic and manual segmentation methods. Notably, hippocampal volumes estimated using automated segmentation techniques demonstrated a strong correlation with those obtained through manual measurements. In light of the limitations identified in automatic segmentation methods, we suggest future research directions focusing on refining these techniques to enhance accuracy and reliability. Integrating advanced machine learning algorithms with traditional manual methods may yield improved segmentation outcomes, thus providing more robust data for clinical applications. Such endeavors could further elucidate the relationship between hippocampal morphology and cognitive function, ultimately advancing our understanding of Alzheimer disease and its progression.

## Acknowledgments

The authors would like to extend their gratitude to Ms. Ardha Ardea Prisilla, Mr. Fahni Harris, Mr. Gilang Titah Ramadhan, and Ms. Maftuhah Rahimah Rum for their assistance.

## Author contributions

**Conceptualization:** Yori Pusparani, Chi-Wen Lung.

**Methodology:** Yori Pusparani, Chih-Yang Lin.

**Supervision:** Fu-Yu Lin, Ben-Yi Liau, John Sahaya Rani Alex, Jeetashree Aparajeeta, Wen-Hung Chao.

**Writing – original draft:** Yori Pusparani.

**Writing – review & editing:** Yih-Kuen Jan, Chi-Wen Lung.

## Supplementary Material
