# Supplementary material for: Hippocampal volume asymmetry in Alzheimer disease: A systematic review and meta-analysis
Source: Medicine (Baltimore). 2025 Mar 7;104(10):e41662. doi: 10.1097/MD.0000000000041662 (PMC11903023; doi:10.1097/MD.0000000000041662)
Supplement: Supplementary file 1 [file medi-104-e41662-s001.docx]

Appendix 1. Search strategy

| Database | Algorithm | | total |
| --- | --- | --- | --- |
| PubMed | (((((("Alzheimer"[Title/Abstract] OR "Alzheimer's"[Title/Abstract]) AND "Hippocampus"[Title/Abstract]) OR "Hippocampal"[Title/Abstract]) AND "Volume"[Title/Abstract]) OR "brain volume"[Title/Abstract] OR "Volumetry"[Title/Abstract]) AND "segmentation"[Title/Abstract]) AND ((fft[Filter]) AND (english[Filter])) | | 1,361 |
|  | |  |  |
|  | |  |  |
| Web of Science | Alzheimer OR Alzheimer's AND Hippocampus OR Hippocampal AND Volume OR Brain Volume OR Volumetry AND segmentation | | 3,081 |
| Scopus | ( TITLE-ABS-KEY ( alzheimer ) OR TITLE-ABS-KEY ( alzheimer's ) AND TITLE-ABS-KEY ( hippocampus ) OR TITLE-ABS-KEY ( hippocampal ) AND TITLE-ABS-KEY ( volume ) OR TITLE-ABS-KEY ( brain AND volume ) OR TITLE-ABS-KEY ( volumetry ) AND TITLE-ABS-KEY ( segmentation ) ) AND ( LIMIT-TO ( DOCTYPE , "ar" ) ) AND ( LIMIT-TO ( LANGUAGE , "English" ) ) | | 353 |
